# Experiences and perceptions of Ghanaian midwives on labour pain and religious beliefs and practices influencing their care of women in labour

**DOI:** 10.1186/s12978-016-0252-7

**Published:** 2016-11-14

**Authors:** Lydia Aziato, Hannah Antwi Ohemeng, Cephas N. Omenyo

**Affiliations:** 1 Department of Adult Health, School of Nursing, College of Health Sciences, University of Ghana, P.O. Box LG 43, Legon, Accra, Ghana; 2 Department of Adult Health, School of Nursing, University of Ghana, Legon, Accra, Ghana; 3 College of Education, University of Ghana, Legon, Accra, Ghana

**Keywords:** Religion, Midwifery practice, Religion, Spirituality, Religious artefacts, Qualitative research

## Abstract

**Background:**

Beliefs surrounding pain during childbirth has biblical foundations that contribute to labour pain being viewed as a natural phenomenon. Contemporary health care promotes evidence-based labour pain management but the faith of the midwife may influence her midwifery practice regarding labour pain management. Therefore this study sought to gain in-depth insight into the experiences and perceptions of midwives regarding labour pain and the religious beliefs and practices influencing their care of women in labour in Ghana.

**Methods:**

The design of the study was an interpretive phenomenology using individual in-depth interviews. The study participants were 27 Ghanaian female midwives of various religious backgrounds. Interviews were conducted in English, audio-taped and transcribed verbatim. Colaizzi’s qualitative analysis procedures were employed concurrently with data collection.

**Results:**

Three major themes were generated: religious beliefs about labour pain, religious practices in labour and religious artefacts used in labour. The midwives’ faith and their experiences during their midwifery practice were inter-connected. The midwives believed labour pain was natural and religious practices are important to prevent complications. Religious artefacts used in labour included anointing oil and water, necklaces, rubber bands, bracelets, stickers and beads.

**Conclusion:**

It is important that midwives provide an enabling environment for women in labour to practice their faith and they should employ context-appropriate strategies to effectively manage labour pain that takes into account the religious beliefs and practices of women.

## Plain English summary

Midwives take care of women who experience labour pain during delivery. Pain associated with labour is considered normal and the faith of the midwife influences her midwifery practice.

We sought to understand midwives’ experiences and perceptions of labour pain and religious beliefs and practices influencing their care of women in labour in Ghana.

We conducted individual interviews among 27 Ghanaian midwives and we asked for clarifications during the interviews as they shared their experiences on religious beliefs and practices. Audio-taped interviews were systematically analyzed and themes identified.

We realized three major findings such as religious beliefs about labour pain, religious practices in labour and religious articles or items used in labour. It was evident that the midwives’ faith and their experiences during their midwifery practice were closely inter-related. The midwives believed labour pain was natural and religious practices are important to prevent complications. Religious articles or items used in labour included anointing oil and water, necklaces, rubber bands, bracelets, stickers and beads.

We concluded that it is important that midwives provide an enabling environment for women in labour to practice their faith and they should employ context-appropriate strategies to effectively manage labour pain that takes into account the religious beliefs and practices of women.

## Background

Midwives manage women in labour at different care settings, including private maternity homes and hospitals. Many females continue to experience varying intensities of labour pain during child birth [1, 2]. Previous researchers note that midwives’ labour pain management is considered inadequate in Ghana [3, 4]. It is known that pregnancy and labour have strong religious connotations among women and that childbearing women exhibit their faith through diverse religious practices and use of religious artefacts to boost their faith [5, 6]. In this regard, the female midwife who also has her own set of religious beliefs and practices is confronted with a complex situation during her practice where women in labour require her support for their unique religious needs [7].

Labour pain is referenced to the Bible verse where God cursed the woman to suffer labour pain when the early man disobeyed God (Genesis 3:16). Labour pain is therefore considered normal by most women and midwives [8, 9]. The perception of labour pain as natural physiological process may influence midwives of the Christian faith who depend on this scripture to expect women to experience labour pain. Some midwives would then reassure women in labour that it is natural to experience labour pain [4, 10]. In a similar vein, a woman in labour would desire to have an uncomplicated labour as stated in the Bible where Hebrew women give birth on their own before the midwives arrived (Exodus 1:19) [11]. Thus, women and midwives in labour would pray for safe childbirth through diverse ways depending on their faith [12]. Women with Islamic faith recite Quran 36: Surah Ya-Sin for safe childbirth [13].

Some individuals believe that pregnancy and labour are associated with evil spirits that pre-dispose women to complicated labour contributing to maternal and neonatal mortality [9, 14, 15]. In an attempt to prevent plots of evil spirits, women employ several religious practices such as prayer during pregnancy and labour [5]. Women from different religious backgrounds use some religious artefacts such as anointing oil (olive oil that is blessed by the religious leader), rosaries, stickers, beads and bands. These are believed to grant further protection [6, 16]. The specific artefact used is usually determined by the religious affiliation of the woman. Midwives may have difficulty accepting the use of certain artefacts if they perceive that such artefacts will interfere with the care they provide and negatively affect the health of the woman. For example, midwives may not allow the use of herbal decoctions or oils during labour especially where there is the possibility of a caesarian birth [17, 18].

The faith of the midwife could interfere with her care delivery in cases where women’s religious practices conflict with those of the midwife and this could result in dissatisfaction of care [19, 20]. For example a midwife of the Christian faith may not permit some traditional practices on the labour ward because such practices are not recommended by her faith [21, 22]. The midwife may also be afraid of some religious artefacts women use in labour and this may interfere with care especially if the woman refuses to remove such artefacts. Again, the midwife’s faith may influence her attitude towards a woman’s demonstration of her faith either through loud prayer or singing [20, 23]. In this instance, the midwife may interfere by asking the woman to be quiet if the practice of the midwife’s faith differs [21].

The interconnections of a midwife’s faith and her midwifery practice can be linked to her gender role as a woman who might have gone through labour pain and applied one religious artefact or the other [16, 21]. Therefore, in this study the female midwife who had used religious artefact during her own pregnancy and childbirth may care for a woman using the same artefact. In Ghana, midwifery training was restricted to women until 2013 when male community health nurses and nurse assistants were admitted into a 2 year post-basic midwifery programme in three midwifery training institutions on pilot basis. After two groups were admitted, the Ministry of Health has halted the admission of male community health nurses for midwifery training. Midwives in Ghana work at all the levels of the health sector and some are in private practice. Midwives engage in autonomous practice but, they refer to gynaecologists or collaborate with other health professionals such as anaesthetists to care for women in labour. Midwives in Ghana are not permitted to perform caesarian births or give epidural analgesia. Therefore, they work closely with gynaecologists especially in cases of complicated pregnancy and labour. They are allowed to prescribe first line drugs used for obstetric care such as haematinics, analgesics and oxytocics.

Previous researchers have investigated labour pain management among midwives [24] and the experiences of women in labour [3, 4]. However, there is inadequate exploration of the influence of the midwives’ faith on labour pain management and the use of religious artefacts. This study sought to gain an in-depth understanding of the experiences and perceptions of midwives and the religious beliefs and practices influencing their care of women in labour. This study is part of an ongoing larger study on the effect of religiosity on pregnancy and on childbirth in Ghana.

## Methods

### Design

The study adopted interpretive phenomenology to investigate the lived experiences of midwives regarding how their faith influences the management of labour pain [16, 25]. This approach of phenomenology allowed the researchers to gain in-depth understanding of the interconnections between the midwives’ faith, religious beliefs and practices and their influence on the care they provided to women in labour. Lived experiences are holistic and inter-related such that it is sometimes difficult to delineate one experience from the other. Therefore, this study presents findings with intersections between the midwives’ perceptions on labour pain and experiences of religious beliefs and practices during care of women in labour.

### Study setting

The study was conducted at the labour wards of a tertiary health facility in Accra, Ghana. The hospital has two labour wards and twenty midwives working in these wards were included. In addition to the midwives on the labour wards, two midwifery educators in Accra and five midwives in Tamale were purposively recruited to develop themes generated in the study.

### Target population and sampling

The target population was religious practising midwives who were willing to take part in the study. Midwives who had worked for a minimum of three years were recruited for the study. The study was explained to all the midwives on the labour wards and those who volunteered and met the inclusion criteria were recruited. At the time of data collection, the first cohort of male midwives were yet to graduate therefore, this study involved only female midwives.

### Data collection procedures

The individual interviews were conducted in English. The interviews lasted 30 to 45 min. Open ended questions were asked to stimulate free individual expressions. Probes were used to gain in-depth understanding of the phenomenon under investigation. The first author (LA) who is experienced in qualitative interviewing collected all the data. The place and time of interviews were at the convenience of the participants. The interviews were audio-recorded with a digital voice recorder with the consent of participants. Twenty-seven interviews were conducted in this study between March and July, 2015. The interviews were transcribed verbatim and field notes were written on context and non-verbal behaviour during the interview. Reflections during data collection were also written as part of field notes to ensure that the views of midwives were represented faithfully.

### Data management and analysis

Concurrent data analysis was undertaken following the processes of qualitative analysis proposed by Collaizzi [26]. Transcripts were read several times to fully understand the religious life of the midwives and how it impacted on their labour pain management. The transcripts were coded and similar codes were grouped. The first and second author coded the data independently and differences were discussed for a consensus on the most appropriate code for a piece of data. Subsequently, themes and sub-themes generated were discussed among the three research team members and any discrepancies were resolved by going back to the data and ensuring that the theme and sub-themes accurately represented the world of the midwives. The data was managed with NVivo software version 10.

### Rigour

Trustworthiness was maintained through a number of processes. For example, one member of the research team collected all the data which ensured that similar questioning techniques were used. Concurrent analysis ensured that themes were fully developed in this study. Member checking (asking participants follow-up questions to confirm themes and sub-themes generated during concurrent analysis) ensured that any gaps in the data were filled and the midwives reviewed the themes generated as a true representation of their world. A detailed audit trail was kept and verbatim quotes gave adequate context that can enhance application of findings in similar settings.

## Results

### Demographic characteristics

Twenty-seven midwives between 25 and 59 years old participated in the study. The midwives included 25 practising midwives and two midwifery educators. Eighteen of them had children ranging from one to three in number and nine of the midwives had no children. Nineteen of the midwives were married, seven were single and one was divorced. The religious background of participants were: 24 Christian and three Muslim. The midwives had worked for a period ranging from three years to 25 years. Their ranks ranged from Staff Midwife to Principal Midwifery Officer. The midwives were all Ghanaians from various ethnic backgrounds such as Akans, Ga, Ewe, Dagao, Gonja, Dagomba and Grusi. These ethnic backgrounds span across the three geographical zones in Ghana where the cultural backgrounds differ. The diverse backgrounds of the midwives led to generation of rich data on labour pain and religiosity.

The study generated three main themes: religious beliefs about labour pain, religious practices in labour and religious artefacts used in labour.

### Religious beliefs about labour pain

The Christian midwives believed that women experienced labour pain because of a curse from God, as indicated in the Bible. The midwives reported that it is natural that women go through pain during childbirth.

*Labour pain is the curse that we received. Men are to work hard before they get what to eat but for us women, we go through difficult deliveries* (Mary)

*It is something that God has already written in the Bible. It is natural* (Aku); *…so once in a while, we tell the mothers about the fact that it was even written in the bible* (Abena)

Some midwives were of the view that it is the same God who gave humans the knowledge to control the pain; so, labour pain should be relieved.

*God has given us the knowledge to control that suffering. So why do we not apply it?… though He said we will suffer through childbirth, it is the same God who has been compassionate unto us by giving us knowledge to be nurses, doctors, midwives and also to make drugs to control pain; so why do we not use it?* (Aba)

The biblical foundation of labour pain and safe childbirth was related to the bible and as such, the midwives prayed for women in labour so God could deliver them safely and with less pain.

*Oh God! help these people to bring the pain down or help them for in the quick dilation of the cervix so that they can deliver and come out of the pain. That is how I feel when they are going through that pain.* (Aba)

The same biblical foundation about God’s intervention was showed in the lyrics of songs women used when experiencing labour pain with the belief that the pain would be relieved.

*The woman had this song; ‘God, if you don’t speak to me, I won’t go anywhere. Lord speak so that my heart will be at peace’. She started singing this song any time she had a contraction.* (Baaba)

In view of this belief that, the faith of the women in labour can reduce their pain and that pain is natural, some midwives left the women in labour to their *fate*. In these instances, the women experienced pain until they gave birth.

*Pain management is very low at times when we are busy on the ward, we just leave them to their fate.* (Maa)

The midwives reported that women shouted that they were in pain even after they prayed or sang suggesting that the prayer and singing did not control the pain.

*Sometimes, she will shout and tell you that even after the prayer and singing, she is still in severe pain. This could mean that the prayer and singing were not effective.* (Sarah)

Also, some midwives believed that a pregnant woman is susceptible to many evil spirits especially in the night and these may cause difficult labour.

*I believe that once you are pregnant, you are susceptible to so many things. You don’t bath in the night because the witches and wizards will enter your body and destroy your pregnancy or cause difficult labour.* (Lizzy)

Thus, the midwives believed that when a woman gives birth to a baby safely after a difficult labour, it is a miracle. *‘I thank God so much when women deliver safely especially a difficult delivery. It is just a miracle’.* (Akos)

A midwife was not allowed to examine a woman in severe labour pain and when the woman lost her baby, the midwife thought that it was the woman’s fault rather than God’s.

*A woman was in severe pain and she did not allow us to examine her and she lost her baby. So she said: ‘God, you have disappointed me’… I thought it was her own disobedience.* (Maa)

Muslim midwives in the study stated that there is a verse in the Holy Quran that specifies safe delivery and they used this verse to pray with the hope of safe childbirth.

*I used to pray with a verse in the Quran referring to safe delivery when I was in labour, but I have forgotten the specific verse.* (Fati)

### Religious practices in labour

All the midwives in this study observed that women prayed when in labour for safe childbirth and pain relief. Some midwives prayed with women in labour when they requested to be prayed for.

*Some pregnant women inquire about your faith and the church you attend. So when she is comfortable with you, she may ask for the prayer’.* (Sarah)

Some midwives allowed pastors to come into the labour ward to pray for the women while others did not allow them because of lack of privacy on the ward and the noise associated with such prayers.

*We once allowed a pastor in and the noise was so much; ….we tell them often: ‘this is an open ward so pray outside the labour ward’.* (Sarah)

Some midwives did not allow women to drink herbal concoctions which the women believed could ease their labour pain.

*…we do not allow women to drink any herbal concoctions because if anything happens to the baby, they will blame us (the midwives)… they believe that when they drink it, the baby will come out quickly and their pain will be better.* (Kate)

Some midwives joined women to participate in radio or television religious programmes on the labour ward.

*…when we go to the ward we have a radio set and TV set. If there is a channel where a pastor is praying, some of the midwives together with the women pay attention and say ‘Amen’ at the end of the prayer.* (Maa)

Some midwives allowed women in labour to drink anointing water but not the anointing oil.

*….I allow the women to drink the anointing water but not the anointing oil; I will ask: ‘can you call your pastor and ask if we can spray it on your abdomen?’ We then do that instead.* (Freda)

A Muslim midwife explained the process of washing Quran verses written on special boards for women who could not recite the verses.

*…the Imams mix and burn sugar. They usually use something like ‘solution board’ on which they write the same verse onto the board using a stick dipped into burnt sugar. After writing the whole verse, they will wash with water. It will now be a solution for the woman to drink so that she goes through pregnancy and labour safely. …If you can recite it yourself, you recite a particular verse every day when you get up. But for those who cannot recite, they wash it for them to drink.* (Fati)

The midwives reiterated the need for spirituality to be added to the midwifery curriculum.

*Adding spirituality to the curriculum will help the midwives to be well informed so that they can understand and help pregnant women and those in labour better.* (Freda)

### Religious artefacts used in labour

Artefacts in this study refers to items or objects used during labour to demonstrate religiosity.

Some midwives asked women to take off necklaces when in labour with the fear of women pulling it when in pain.

*For those with necklaces, we tell them to take it off because when they are in pain, some of them hold it. Because they don’t know what is going on, they can hold it and suffocate. …I have not encountered anyone who has ever disagreed. But in the case of going to the theatre, or during labour, we insist they take it off but if the woman refuses, we don’t touch it.* (Bless)

A midwife advised a woman in labour who was watching the pastor’s picture to put it away and asked her to pray on her own. The woman later lost her uterus and baby on account of ruptured uterus and the midwife was unable to visit the woman because she felt guilty.

*I saw a woman in labour watching a picture of her pastor… I advised her to pray for herself and she stopped looking at the picture… The next day when I reported to work, I was told they suspected ruptured uterus and she was sent to the theatre. And unfortunately after the total hysterectomy, she lost the baby also… In fact I didn’t even want the woman to see my face because she may think I am a devil. So since then, I am careful with the spiritual items of women*. (Angie)

## Discussion

It was realized that midwives related labour pains to the Bible and believed that God could help women go through uncomplicated labour and safe delivery. They also believed that pregnancy and labour were associated with evil spirits. Therefore, both midwives and women in labour prayed.

Midwives’ beliefs about labour pain in this study are in tandem with those of previous researchers especially the biblical references of labour pain and uncomplicated birth [8, 9, 24]. The phenomenon of God helping to ensure a safe childbirth was common to all religious affiliations in this study including those of the Islamic faith. Therefore, to connect with God, women in labour prayed through diverse ways and requested prayer support from their pastors. These findings are similar to other studies [5, 9, 16, 27]. However, some midwives asked pastors who were noisy in their prayers to tone down and others did not allow pastors into the labour ward in an attempt to protect the privacy of other women. This finding indicates that although midwives acknowledge the role of prayers in labour, they are also conscious of individual differences so that one woman would not inconvenience other women in labour [28]. Also, in shared or open labour wards, the midwives did not compromise the privacy of other women to meet the religious needs of another [29]. Some midwives convinced women in labour who demonstrated faith in their pastors by viewing their pictures to rather have faith in God for a successful labour. In such instances of interference of the women’s faith, the midwives felt guilty upon a negative outcome of labour. The midwife’s feeling of guilt and inability to follow-up on women after complications of labour showed that midwives’ faith and practice are a complex whole where one cannot rule out religiosity in the care of pregnant women. Faith is abstract and personal [30]; therefore midwives should appreciate the faith of pregnant women and those in labour so that their care will be rendered within the standards of care in the hospital setting [31]. It is necessary for midwives to be cautious in interfering with the faith or religious practice of women in labour so that this feeling of guilt will be prevented and midwives can continue to follow-up on their women after childbirth [3, 32]. However, in most cases, midwives in this study were able to accommodate the religious beliefs of women and the women also cooperated so that care received will not be hindered.

However, some midwives in this study reported on fear associated with some religious artefacts which could interfere with their professional practice. These suggest that midwives should find out the religious artefacts within their practice areas so that when women use them during labour, they will not be afraid. Again, midwives should accommodate women who use artefacts and allow them privacy and personal space to observe their religious practice during labour.

The diverse religious backgrounds of the midwives enhanced a full understanding of the midwives’ experiences. However, similar to all qualitative studies, the small sample size limits generalization of the findings. Data saturation was achieved when no new findings were generated from the interviews. The inadequate number of Muslim midwives in this study could be a limitation since involvement of more Muslim midwives could generate new findings. Future studies could include other religious sects to corroborate the findings in this study.

## Conclusion

The findings in this study demonstrate the need for an update of the curriculum to ensure teaching midwifery students issues of spirituality as recommended by a study participant in this study. The curriculum upgrade will contribute to midwives being well informed about the spiritual needs of pregnant women and those in labour. Pastors should be educated on pregnancy and labour process and the pain the women experience so that they can support pregnant women better. It is necessary for the Ghana Health Service to formulate a policy that would provide women the supportive environment to meet their spiritual needs rather than go to the prayer camps. Midwives should ensure that their own faith do not interfere with labour pain management [21, 32].

Religiosity is an integral part of child birth due to the popular belief that pregnancy and labour are influenced by evil forces. Also, labour pain is seen as a natural phenomenon and midwives should adequately manage the pain. We anticipate that enhanced education of midwives and women would ensure that both parties collaborate during labour for women to meet their religious needs and midwives not practice in conflict with their personal faith. Such education should be done during pregnancy for women and during training for midwives. The midwives’ understanding of the women’s worldview would contribute immensely to cooperation during care. It is important that religious artefacts used in labour do not interfere with professional care received. Therefore midwives should be sensitive in handling issues of religious artefacts to ensure satisfaction for both parties.

## References

[1] Beigi NMA, Broumandfar K, Bahadoran P, Abedi HA (2010). Women’s experience of pain during childbirth. Iranian J Nurs Midwifery Res.

[2] Veringa I, Buitendijk S, de Miranda E, de Wolf S, Spinhoven P (2011). Pain cognitions as predictors of the request for pain relief during the first stage of labor: a prospective study. J. Psychosom. Obstet. Gynaecol..

[3] Mensah RS, Mogale RS, Richter MS (2014). Birthing experiences of Ghanaian women in 37th Military Hospital, Accra, Ghana. Int J Africa Nurs Sci.

[4] D'Ambruoso L, Abbey M, Hussein J (2005). Please understand when I cry out in pain: women's accounts of maternity services during labour and delivery in Ghana. BMC Public Health.

[5] Ozsoy SA, Katabi V (2008). A comparison of traditional practices used in pregnancy, labour and the postpartum period among women in Turkey and Iran. Midwifery.

[6] Fouka G, Plakas S, Taket A, Boudioni M, Dandoulakis M (2012). Health-related religious rituals of the Greek Orthodox Church: their uptake and meanings. J Nurs Manag.

[7] Timmins F, Neill F (2013). Teaching nursing students about spiritual care; A review of the literature. Nurse Educ Pract.

[8] Halperin O, Sarid O, Cwikel J (2014). A comparison of Israeli Jewish and Arab women's birth perceptions. Midwifery.

[9] Kaphle S, Hancock H, Newman LA (2013). Childbirth traditions and cultural perceptions of safety in Nepal: critical spaces to ensure the survival of mothers and newborns in remote mountain villages. Midwifery.

[10] Abushaikha L, Oweis A (2005). Labour pain experience and intensity: a Jordanian perspective. Int J Nurs Pract.

[11] Ellis E (1873). Biblical Obstetrics. Lancet.

[12] Callister LC, Khalaf I (2010). Spirituality in Childbearing Women. J. Perinat. Educ..

[13] Ahmadi Z (2013). Positive experiences of childbirth: a phenomenological study. Researcher.

[14] Maimbolwa MC, Yamba B, Diwan V, Ransjo-Arvidson AB (2003). Cultural childbirth practices and beliefs in Zambia. J Adv Nurs.

[15] Ngomane S, Mulaudzi FM (2012). Indigenous beliefs and practices that influence the delayed attendance of antenatal clinics by women in the Bohlabelo district in Limpopo, South Africa. Midwifery.

[16] Aziato L, Odai P, Omenyo C (2016). Religious beliefs and practices in pregnancy and labour: an inductive qualitative study among post-partum women in Ghana. BMC Pregnancy Childbirth.

[17] Lamxay V, de Boer HJ, Björk L (2011). Traditions and plant use during pregnancy, childbirth and postpartum recovery by the Kry ethnic group in Lao PDR. J Ethnobiol Ethnomed.

[18] Hunter LP (2006). Women give birth and pizzas are delivered: language and Western childbirth paradigms. J Midwifery Womens Health.

[19] Litoff JB (1982). The Midwife Throughout History. J Nurse Midwifery.

[20] Linhares CH, Manoa UH. Mana from heaven: The essential structure of the lived experiences of nurse-midwives with the concept of spirituality in childbirth. A Phenomenological Study. University of Hawai'i at Manoa; 2007. Retrieved from https://scholarspace.manoa.hawaii.edu/handle/10125/22059.

[21] Linhares CH (2012). The lived experiences of midwives with spirituality in childbirth: mana from heaven. J Midwifery Womens Health.

[22] Roseghini M, Olson S (2015). What do midwives think about midwifery supervision?. Bri J Midwifery.

[23] Russell KE (2007). Mad, bad or different? Midwives and normal birth in obstetric led units. Br J Midwifery.

[24] Ampofo EA, Caine V (2015). A narrative inquiry into women’s perception and experience of labour pain: A study in the western region of ghana. Int J Africa Nurs Sci.

[25] de Vos AS, Strydom H, Fouché CB, Delport CSL (2011). Research at Grass Roots: For the Social Sciences and Human Service Professions.

[26] Shosha GA (2012). Employment of Colaizzi's strategy in descriptive phenomenology: A reflection of a researcher. European Sci J.

[27] Wulandari LP, Klinken Whelan A (2011). Beliefs, attitudes and behaviours of pregnant women in Bali. Midwifery.

[28] Redshaw M, Heikkila K (2011). Ethnic differences in women's worries about labour and birth. Ethn Health.

[29] Bonilla-Escobar FJ, Ortega-Lenis D, Rojas-Mirquez JC, Ortega-Loubon C (2016). Panamanian women׳s experience of vaginal examination in labour: A questionnaire validation. Midwifery.

[30] LaVigne B (2013). A Personal Perspective On Faith versus Religion. Verbum.

[31] Al Husseini DI. The implications of religious beliefs on medical and patient care. University of Pennsylvania; 2011. Retrieved from http://repository.upenn.edu/cgi/viewcontent.cgi?article=1047&context=od_theses_msod.

[32] Johnson MR (2004). Faith, prayer, and religious observances. Clin Cornerstone.

